# Venous thromboembolism in Cushing syndrome: current evidence and a practical thromboprophylaxis algorithm

**DOI:** 10.3389/fendo.2026.1927311

**Published:** 2026-09-29

**Authors:** Paweł Kuca, Paulina Kostrzewska, Bartosz Migda, Przemysław Witek

**Affiliations:** 1 Department of Internal Medicine, Endocrinology and Diabetes, Mazovian Brodnowski Hospital, Warsaw, Poland; 2 Department of Internal Medicine, Endocrinology and Diabetes, Medical University of Warsaw, Warsaw, Poland; 3 Diagnostic Ultrasound Lab, Department of Pediatric Radiology, Medical University of Warsaw, Warsaw, Poland

**Keywords:** Cushing syndrome, fibrinolysis, hypercoagulability, hypercortisolism, thromboprophylaxis, venous thromboembolism

## Abstract

Cushing syndrome (CS) is a rare endocrine disorder associated with a markedly increased risk of venous thromboembolism (VTE). Chronic hypercortisolism induces a prothrombotic state through activation of coagulation pathways, impaired fibrinolysis, and endothelial dysfunction. VTE represents a major source of morbidity and mortality in this population, yet optimal strategies for prevention and treatment remain insufficiently standardized. To summarize current evidence on the epidemiology, pathophysiology, risk factors, prevention, and treatment of VTE in patients with endogenous CS and to propose a practical thromboprophylaxis approach. A narrative review of the literature was conducted using MEDLINE/PubMed, Scopus, and Embase databases. Observational studies, registry data, systematic reviews, and expert consensus statements addressing VTE in CS were analyzed, with particular attention to peri-operative risk and persistence of hypercoagulability after remission. Patients with CS have a 10–20-fold higher risk of VTE compared with the general population. The peri-operative period, especially after TSS or adrenalectomy, represents the highest-risk phase, although increased thrombotic risk may persist for 3–6 months after biochemical remission. Key mechanisms include elevated factor VIII, von Willebrand factor, plasminogen activator inhibitor-1, increased thrombin generation, and endothelial dysfunction. Recent evidence from the ERCUSYN registry and the 2025 Delphi consensus supports early and extended thromboprophylaxis. Current expert consensus supports low-molecular-weight heparin as the preferred peri-operative pharmacological agent, whereas direct oral anticoagulants may be considered in selected patients during long-term management. CS should be regarded as a persistent prothrombotic condition in which early and, when appropriate, extended thromboprophylaxis should be considered following individualized assessment of thrombotic and bleeding risk. Prospective studies are needed to develop evidence-based, individualized prevention strategies.

## 1 Introduction

Cushing syndrome (CS) is a rare but severe endocrine disorder characterized by chronic exposure to excessive glucocorticoids, either endogenous or exogenous. The estimated annual incidence of endogenous CS ranges from 0.7 to 2.4 cases per million inhabitants (1).

The hypercortisolemic state leads to profound metabolic, cardiovascular, musculoskeletal and immunologic consequences (2, 37). Among its less frequently discussed but clinically significant complications are a markedly increased risk of VTE, including deep vein thrombosis (DVT) and pulmonary embolism (PE). Several cohort and registry studies have demonstrated that patients with CS exhibit a VTE incidence up to 10–20 times higher than that observed in the general population (3–5). The risk is particularly elevated in the peri-operative period following transsphenoidal surgery (TSS) or adrenalectomy, but persists for weeks to months after biochemical remission (6, 7). Mechanistically, hypercortisolism induces a pro-coagulant and hypofibrinolytic state, involving elevations in factor VIII (FVIII), von Willebrand factor (vWF), and plasminogen activator inhibitor-1 (PAI-1), along with increased thrombin generation and endothelial dysfunction (8, 9). Despite growing evidence, no standardized recommendations currently exist regarding the timing, duration, or intensity of thromboprophylaxis in CS. Clinical decisions therefore rely largely on expert opinion, small series, and extrapolation from non-CS populations (9). Given the potentially fatal nature of VTE and the absence of unified guidelines, a comprehensive synthesis of existing data is warranted. This review aims to summarize the current knowledge on the epidemiology and timing of VTE in CS, underlying pro-thrombotic mechanisms, established and emerging risk factors, current recommendations for prevention and management and existing research gaps.

## 2 Pathophysiology of hypercoagulability in CS

The hypercoagulable state observed in CS is multifactorial and arises from complex interactions between coagulation, fibrinolysis, inflammation, and endothelial function. Chronic exposure to excessive glucocorticoids promotes a prothrombotic milieu that persists even after biochemical remission in many patients (3, 8).

### 2.1 Activation of coagulation pathways

Several studies have demonstrated elevated plasma concentrations of FVIII and vWF in patients with active CS (4, 10, 11). These factors, both produced or stored in endothelial cells, reflect an activated endothelium and play a central role in platelet adhesion and clot propagation.

Increased fibrinogen and thrombin–antithrombin complexes (TAT) further indicate accelerated thrombin generation (4). Importantly, these abnormalities are not immediately reversible following remission; short-term normalization of cortisol levels does not restore hemostatic balance (8).

### 2.2 Impaired fibrinolysis

The fibrinolytic system is significantly inhibited in CS. Elevated PAI-1, together with reduced tissue plasminogen activator (tPA) activity, leads to impaired fibrin degradation and prolonged clot persistence (12). PAI-1 overexpression is driven by glucocorticoid-induced up-regulation of SERPINE1 gene transcription and by insulin resistance, central obesity, and dyslipidemia—common metabolic features of CS (13).

### 2.3 Endothelial dysfunction and inflammation

Hypercortisolism contributes to endothelial injury through oxidative stress, increased expression of adhesion molecules (ICAM-1, VCAM-1), and reduced nitric-oxide bioavailability (14). These changes, combined with low-grade systemic inflammation, potentiate both platelet activation and coagulation cascade amplification. Elevated circulating levels of interleukin-6 (IL-6) and C-reactive protein (CRP) correlate with disease activity and pro-coagulant marker concentrations (15).

### 2.4 Platelet activation and glucocorticoid-mediated effects

Glucocorticoids directly influence platelet reactivity by enhancing thromboxane A_2_ synthesis and surface receptor expression (e.g., glycoprotein IIb/IIIa). Hypercortisolism also promotes erythrocytosis and plasma viscosity, further favoring stasis (16).

### 2.5 Persistence after remission

Although surgical remission of hypercortisolism improves metabolic parameters, a subset of patients retains laboratory evidence of hypercoagulability months after surgery (8, 10). This delayed normalization likely explains the continued elevated risk of VTE observed up to 3–6 months post-remission (9).

Collectively, these findings indicate that CS-associated hypercoagulability is a composite phenomenon driven by increased pro-coagulant activity, impaired fibrinolysis, and endothelial dysfunction, providing a biological rationale for extended thromboprophylaxis beyond the acute peri-operative period.

## 3 Epidemiology and timing of VTE in CS

VTE represents a major cause of morbidity and mortality in patients with CS. The prevalence and incidence of thromboembolic complications in CS vary widely between studies, largely due to heterogeneity in patient populations, diagnostic methods, and timing of follow-up. Nonetheless, nearly all available data confirm that CS is associated with a markedly increased risk of VTE compared with the general population (3–5).

### 3.1 Incidence and prevalence

Early single-center cohorts estimated that 2–8% of patients with active CS develop clinically apparent VTE (7, 17). More recent multicenter and registry-based analyses have refined this estimate. In the large European ERCUSYN registry (n = ≈1500), 7.7% of patients experienced VTE during the disease course, corresponding to an incidence rate of 14.6 events per 1000 person-years, nearly 10–20-fold higher than in matched controls (5). Meta-analytic data confirm this excess risk, reporting a pooled odds ratio (OR) of ~18 for VTE in CS compared to the general population (8). The elevated risk is consistent across ACTH-dependent and ACTH-independent subtypes, though it may be slightly higher in patients with ectopic ACTH secretion.

### 3.2 Timing of thromboembolic events

The highest risk period for VTE occurs around the time of surgery, particularly TSS for Cushing’s disease and adrenalectomy for adrenal CS (5–7).

Peri-operative studies have documented rates of postoperative VTE up to 10–20% when systematic imaging (e.g., duplex ultrasonography) is employed, with a substantial proportion of asymptomatic DVT detected (7, 18).

Importantly, observational data indicate that the risk does not normalize immediately after surgical remission; hypercoagulability may persist for weeks to months (8, 9). White et al. reported that most VTEs occur within the first 6 weeks after surgery, yet cases have been documented up to 3 months postoperatively, suggesting a “long tail” of risk (6, 18).

### 3.3 Risk before and after treatment

Thrombotic events can also occur prior to diagnosis, especially in patients with prolonged unrecognized hypercortisolism, where the combination of obesity, immobility, and severe metabolic derangements amplifies the risk (19).

After biochemical cure, the risk declines but remains above baseline for at least several months, in parallel with delayed normalization of fibrinolytic parameters (8).

These findings underscore the need for early recognition and proactive thromboprophylaxis, initiated at diagnosis or preoperatively rather than only postoperatively.

## 4 Peri-operative management: screening and thromboprophylaxis

### 4.1 Rationale and timing

Peri-operative management of VTE risk in CS remains one of the most critical yet inconsistently addressed aspects of care.

The combination of surgical stress, immobilization, and sustained hypercortisolism creates a period of exceptionally high thrombotic risk, particularly in the first 4–6 weeks after surgery (4, 6, 7).

Given the persistence of hypercoagulability after biochemical remission, standard thromboprophylaxis protocols applied in general endocrine or neurosurgical settings are likely insufficient for this population (8).

### 4.2 Pre-operative screening

Pre-operative evaluation for subclinical DVT has been proposed for patients with severe hypercortisolism or additional risk factors (e.g., obesity, previous VTE, limited mobility).

Duplex ultrasonography of lower extremity veins may identify asymptomatic DVT, which occurs in up to 8–10% of patients prior to TSS when systematic screening is performed (17, 18).

Although universal pre-operative screening is not yet recommended in guidelines, may be considered in high-risk individuals according to current expert consensus (9).

### 4.3 Mechanical prophylaxis

Mechanical thromboprophylaxis should generally be initiated before surgery and continued until full mobilization. These measures are especially important during the diagnostic and peri-operative phases when pharmacologic prophylaxis may be temporarily interrupted due to bleeding risk (20).

### 4.4 Pharmacologic prophylaxis

Pharmacologic thromboprophylaxis is considered a cornerstone of peri-operative management in patients with endogenous Cushing’s syndrome. Low-molecular-weight heparin (LMWH) is the most commonly used agent, with dosing typically following standard surgical protocols (e.g., enoxaparin 40 mg s.c. daily or dalteparin 5000 IU s.c. daily). However, due to the prolonged hypercoagulable state, the Delphi consensus (2025) recommends initiating prophylaxis at diagnosis or at least pre-operatively, continuing throughout hospitalization, and extending it for 4–12 weeks post-discharge (9, 21). In selected high-risk patients—those with morbid obesity, previous VTE, markedly elevated FVIII or PAI-1—extended prophylaxis up to 3 months may be justified (5, 22). The use of direct oral anticoagulants (DOACs) as prophylactic agents in CS is under investigation; data remain limited, and LMWH remains preferred in the peri-operative period (21).

Despite the well-established thrombotic risk in patients with endogenous Cushing’s syndrome, the decision to initiate pharmacological thromboprophylaxis should always be balanced against the potential risk of postoperative bleeding, particularly following transsphenoidal surgery. Although clinically significant hemorrhagic complications after TSS are uncommon, postoperative sellar hematoma may lead to neurological deterioration, visual impairment, or the need for urgent surgical re-exploration. Therefore, the timing of anticoagulant initiation should be individualized according to the patient’s postoperative status, surgical hemostasis, and overall bleeding risk. Close collaboration between endocrinologists, neurosurgeons, and thrombosis specialists is essential to optimize the balance between preventing venous thromboembolism and minimizing bleeding complications. Current expert consensus favors early initiation of thromboprophylaxis once adequate hemostasis has been achieved; however, prospective studies evaluating the optimal timing and safety of anticoagulation in this specific population remain lacking (4, 6, 18).

### 4.5 Post-discharge prophylaxis and follow-up

Observational data demonstrate that nearly half of VTEs occur after hospital discharge, most within 6 weeks post-surgery (5). Continuation of LMWH or transition to a DOAC for up to 6–12 weeks may be considered in moderate- to high-risk patients following individualized assessment of thrombotic and bleeding risk (22). Follow-up should include clinical evaluation for signs of thrombosis and, when indicated, D-dimer measurement or ultrasound reassessment.

## 5 Treatment of VTE in CS

### 5.1 General principles

Once VTE is diagnosed in a patient with CS, standard anticoagulant therapy should be initiated promptly, as in the general population. However, CS presents unique challenges related to persistent hypercoagulability, drug interactions, and uncertain optimal duration of therapy (5, 7, 8).

Both the acute phase of hypercortisolism and the peri-remission period are associated with sustained thrombotic risk; therefore, individualized management is essential.

### 5.2 Choice of anticoagulant

Current evidence and expert consensus support LMWH as the preferred initial anticoagulant in hospitalized or peri-operative patients (4). Vitamin K antagonists (VKAs) such as warfarin are effective but less frequently used because hypercortisolism and glucocorticoid therapy alter hepatic metabolism and vitamin K–dependent factor synthesis, complicating INR control (5).

DOACs — particularly apixaban, rivaroxaban, and edoxaban — are increasingly used in stable outpatients after remission or in long-term follow-up (20).

Small real-world series (e.g., Feelders 2024) have shown comparable efficacy and safety of DOACs to LMWH/VKA in CS, though evidence remains limited and mainly retrospective (20, 22).

### 5.3 Duration of anticoagulation

The optimal duration of anticoagulant therapy in CS is not firmly established. Current practice extrapolates from guidelines for provoked VTE with persistent risk factors.

The Delphi consensus 2025 recommends a minimum of 3 months of anticoagulation, with extension to 6 months or longer if laboratory markers of hypercoagulability (e.g., FVIII, vWF, PAI-1) remain elevated or if remission has not been achieved (9).

In selected high-risk individuals — those with recurrent events, severe ACTH-dependent CS, or delayed biochemical cure — indefinite secondary prophylaxis may be considered (9, 19).

### 5.4 Transition and monitoring

Switching from LMWH to a DOAC or VKA can be considered after clinical stabilization and confirmation of hemostatic safety. Regular monitoring may include renal and hepatic function, cortisol status, and selected coagulation markers, depending on the clinical scenario.

Persistent elevation of FVIII > 150 IU/dL or vWF > 175% has been proposed as a surrogate marker of ongoing risk (23).

### 5.5 Glucocorticoid interactions

Pharmacologic interactions may occur between anticoagulants and glucocorticoids:

- Dexamethasone induces CYP3A4, potentially reducing DOAC plasma levels (24).
- Prednisolone may enhance gastrointestinal bleeding risk when combined with VKAs or DOACs (25).
- Rapid withdrawal of glucocorticoids can transiently lower hepatic clearance, altering INR or DOAC exposure (24, 25).

Careful therapeutic monitoring should be considered when clinically indicated.

### 5.6 Treatment after remission

After biochemical remission and normalization of coagulation parameters, most patients can discontinue anticoagulation after 3–6 months, provided there is no recurrence of hypercortisolism or other risk factors (9, 19). Nevertheless, some evidence suggests persistent endothelial activation months after cure, supporting periodic reassessment of D-dimer and FVIII levels before discontinuation (23).

## 6 Exogenous glucocorticoids and risk of VTE

### 6.1 Background

Exogenous glucocorticoid (GC) therapy, widely prescribed for inflammatory, autoimmune, and pulmonary diseases, reproduces several metabolic and hemostatic abnormalities observed in endogenous CS. Accumulating epidemiological evidence demonstrates that GC exposure is associated with an increased risk of VTE, providing a “biological mirror” of hypercortisolism-induced hypercoagulability (26).

### 6.2 Epidemiological evidence

Large population-based studies consistently report a 2- to 3-fold elevation in VTE risk during periods of systemic GC use. A pivotal case-control study from the United Kingdom involving >38–000 VTE cases found that current oral GC users had an adjusted odds ratio (aOR) of 2.3 for first-ever VTE compared with non-users; risk peaked during the first 30 days of treatment and declined after discontinuation (26). Subsequent data from the Danish national registry confirmed these findings: the hazard ratio (HR) for VTE within 90 days of GC initiation was 2.2, increasing up to 3.0 for high-dose regimens (>20 mg prednisolone-equivalent/day) (26).

Short courses of oral corticosteroids — typically defined as bursts lasting up to 14 days — have been associated with a transient increase in the risk of serious adverse events, including VTE, with population-based data showing that even brief OCS exposure (<30 days) can significantly elevate thrombotic risk, particularly in older or vulnerable patients (27, 28).

### 6.3 Dose and duration dependency

The risk of VTE correlates with both dose and cumulative exposure. Chronic users with >3 months of therapy exhibit persistently elevated fibrinogen and PAI-1 levels, whereas short bursts mainly trigger transient endothelial activation and platelet hyperreactivity (29).

Observational data indicate a dose-dependent increase in VTE risk with systemic glucocorticoid use, with higher cumulative prednisolone-equivalent doses being associated with substantially elevated VTE risk, particularly in current users (26, 30).

Inhaled or topical GCs, by contrast, show no consistent association with VTE, underscoring the systemic nature of the prothrombotic effect (26).

### 6.4 Mechanistic parallels with endogenous hypercortisolism

Exogenous GC therapy reproduces several hallmark features of CS-related hypercoagulability:

- increased FVIII and vWF levels,
- suppression of fibrinolysis via up-regulation of PAI-1,
- endothelial activation and reduced nitric oxide bioavailability,
- Venhanced platelet activation and thromboxane A_2_ synthesis (10, 29).

These changes occur even at moderate pharmacological doses and resolve gradually after cessation of therapy.

### 6.5 Clinical implications

From a clinical standpoint, the evidence supports heightened vigilance for thromboembolic events during and shortly after systemic GC therapy. Patients with additional risk factors — obesity, cancer, recent surgery, immobility, or known thrombophilia — may warrant temporary thromboprophylaxis if receiving high-dose or prolonged GC courses (26).

However, routine anticoagulation is not recommended for all GC users due to the short duration of elevated risk and potential bleeding complications (8).

## 7 Controversies and research gaps

### 7.1 Lack of prospective studies and randomized trials

Despite accumulating observational evidence, the management of VTE risk in CS remains largely guided by expert opinion. No randomized controlled trials (RCTs) have evaluated different thromboprophylaxis regimens, drug classes, or durations in CS (7, 8).

Most available studies are retrospective or registry-based, with small sample sizes and heterogeneous endpoints. Consequently, the optimal timing, dose, and duration of prophylaxis — particularly post-remission — remain uncertain (9).

### 7.2 Heterogeneity in study design and outcome definitions

Published cohorts vary widely in the definition of VTE (symptomatic vs screening-detected), inclusion of superficial thromboses, and diagnostic methods (ultrasound vs CT vs clinical suspicion) (6). Many earlier studies excluded asymptomatic events, potentially underestimating the true burden of thromboembolism. Even in large datasets such as ERCUSYN, VTE reporting is inconsistent, and missing data limit subgroup analyses (5, 31).

### 7.3 Limited understanding of post-remission risk

Another unresolved issue concerns the duration of residual hypercoagulability following biochemical cure. While several reports suggest persistence for 3–6 months, longitudinal data beyond one year are lacking (8, 10, 32). It remains unclear whether normalization of cortisol alone is sufficient to reverse procoagulant changes or if long-standing endothelial damage perpetuates risk (33). Defining this window is essential for designing rational prophylactic strategies.

### 7.4 Absence of validated risk-stratification tools

Although Zilio et al. proposed the CS-VTE score based on clinical and laboratory predictors, the model was derived from a single-center cohort and has not undergone external validation. Consequently, no validated risk assessment tool is currently available for routine clinical practice (34). Existing scales (e.g., Padua, Caprini) underestimate risk because they do not incorporate endocrine-specific variables such as serum cortisol, ACTH levels, PAI-1, or FVIII activity (3). Development of a CS-specific scoring system integrating clinical, biochemical, and imaging markers is a major unmet need.

### 7.5 Underrepresentation of special populations

Data in pediatric, elderly, and ectopic ACTH subgroups are extremely limited (5, 35).

Similarly, sex-specific differences and the impact of estrogen therapy or oral contraceptives in women with CS have not been systematically explored.

Pregnant women with CS are virtually absent from existing cohorts despite the compounded thrombotic risk (19).

### 7.6 Need for standardized management algorithms

Although the Delphi consensus (2025) represents an important milestone, its recommendations are based primarily on expert opinion rather than empirical validation (9, 22).

Prospective multicenter studies integrating biochemical monitoring, thrombin generation assays, and imaging surveillance are urgently needed to establish evidence-based protocols.

### 7.7 Existing algorithms for VTE risk assessment and thromboprophylaxis in Cushing’s syndrome

Recent years have witnessed important progress toward the development of structured approaches for venous thromboembolism (VTE) prevention in patients with endogenous Cushing’s syndrome. In 2024, Bryk-Wiązania et al. proposed a practical clinical framework for thrombotic risk assessment, emphasizing the importance of individualized evaluation based on disease activity, patient-related risk factors, and planned surgical intervention (36). Their algorithm provides clinicians with a structured approach to identifying patients who may benefit from thromboprophylaxis while highlighting the limited evidence currently available for many clinical decisions.

Subsequently, the 2025 Delphi consensus statement represented the first international expert consensus specifically addressing thromboprophylaxis in endogenous Cushing’s syndrome. The document provided recommendations regarding perioperative pharmacological prophylaxis, mechanical prevention strategies, timing of anticoagulation, and the potential need for extended prophylaxis following remission. Importantly, the authors acknowledged that most recommendations were based on observational studies and expert opinion, reflecting the absence of randomized clinical trials in this field (9).

The algorithm proposed in the present review is intended to complement rather than replace these previously published approaches. While incorporating the key recommendations of the Delphi consensus, it integrates current evidence regarding the temporal evolution of thrombotic risk, persistence of hypercoagulability after biochemical remission, laboratory biomarkers associated with coagulation activation, and individualized reassessment during follow-up. By combining mechanistic insights with practical clinical decision-making, the proposed algorithm aims to provide a comprehensive framework that may facilitate risk stratification and support individualized thromboprophylaxis until higher-quality evidence becomes available. A comparison of the currently available approaches to VTE risk assessment and thromboprophylaxis in CS is presented in **Table 1**.

**Table 1:** Comparison of published approaches to VTE risk assessment and thromboprophylaxis in endogenous Cushing’s syndrome.

Feature	Bryk-Wiązania et al. (36)	Isand et al. – Delphi Consensus (9)	Present review
Type and scope of approach	Literature-based practical algorithm focused primarily on thrombotic risk assessment outside the surgical setting.	International Delphi consensus providing recommendations for VTE thromboprophylaxis in endogenous CS.	Evidence-informed clinical algorithm integrating available literature and contemporary expert recommendations.
Main inputs / criteria considered	Padua Prediction Score; UFC; CS-VTE score; clinical VTE probability; aPTT and D-dimer; IMPROVE bleeding risk; suspected/confirmed VTE; CS subtype, including EAS and malignancy/ACC; diagnostic imaging (CTPA, V/Q scan, and venous ultrasonography).	Disease activity/biochemical control; previous/family VTE; age >60 years; obesity; immobility; malignancy; thrombophilia; comorbidities; smoking; dehydration; estrogen exposure; pregnancy/postpartum; varicose veins with phlebitis; hospitalization; IPSS; surgery; contraindications to anticoagulation.	Disease activity; previous VTE; age; obesity; immobility; cardiovascular/metabolic comorbidities; surgical status; selected haemostatic biomarkers; individual bleeding risk.
Individual VTE risk assessment	Addressed through stepwise assessment incorporating general and CS-specific risk factors.	Addressed.	Addressed.
Bleeding risk assessment	Addressed; incorporates the IMPROVE bleeding risk score and selected major bleeding-risk factors.	Addressed; contraindications and individual bleeding risk are considered.	Addressed; individualized assessment of thrombotic versus bleeding risk.
LMWH thromboprophylaxis	LMWH is the mainstay of thromboprophylaxis; bleeding risk guides pharmacological versus mechanical prophylaxis. Dose adjustment and anti-Xa monitoring may be considered in selected patients; platelet monitoring is considered when HIT risk is relevant.	Preferred pharmacological approach; standard weight-based prophylactic dosing is recommended, with individual adjustment when appropriate.	Preferred peri-operative pharmacological approach based on current evidence and expert consensus; timing and duration are individualized according to thrombotic and bleeding risk.
Mechanical thromboprophylaxis	Considered in patients at high VTE risk when pharmacological prophylaxis is precluded by high bleeding risk; applicability may be limited by skin fragility in CS.	Addressed; routine antiembolic stockings are not recommended, while mechanical methods may be considered when pharmacological prophylaxis is contraindicated.	Considered according to individual thrombotic and bleeding risk, particularly when pharmacological prophylaxis is contraindicated.
Peri-operative management	Not within the primary scope of the article.	Addressed.	Addressed.
Extended/post-remission thromboprophylaxis	Not within the primary scope of the article.	Thromboprophylaxis is preferably continued for 3 months after biochemical remission, provided there are no obvious contraindications; optimal duration remains uncertain.	Duration individualized according to persistent thrombotic and bleeding risk.
Haemostatic parameters in clinical decision-making	Addressed; aPTT and D-dimer are incorporated into thrombotic risk assessment/diagnostic pathways, while platelet count and anti-Xa activity are considered for safety and LMWH monitoring in selected patients.	Postoperative haemostatic testing is not recommended to guide clinical decision-making.	Selected haemostatic biomarkers are discussed as potential adjuncts to clinical assessment; their utility for guiding prophylaxis remains unvalidated.
Direct oral anticoagulants (DOACs/NOACs)	Not reported as routine thromboprophylaxis in CS. In cancer-related VTE, NOACs may be considered as an alternative to LMWH after assessment of bleeding risk, drug–drug interactions, and patient factors; CS-specific evidence remains limited.	Evidence is insufficient to support DOACs as routine thromboprophylaxis in CS; LMWH remains preferred. The role of DOACs requires further study.	May be considered in selected patients for long-term VTE treatment after assessment of bleeding risk, renal/hepatic function, and drug–drug interactions; evidence for prophylactic use in CS remains insufficient.
Patients with biochemical control during medical therapy	Considered within non-surgical thrombotic risk assessment.	Thromboprophylaxis may not be required in well-controlled patients without additional VTE risk factors.	Individualized reassessment according to biochemical control and additional VTE risk factors.
Prospective/external validation	Proposed algorithm has not undergone prospective external validation.	Not applicable – consensus-based recommendation framework.	Proposed algorithm has not undergone prospective validation.

ACC, adrenocortical carcinoma; aPTT, activated partial thromboplastin time; CS, Cushing’s syndrome; CS-VTE, Cushing’s syndrome–specific VTE risk score; CTPA, computed tomography pulmonary angiography; DOAC, direct oral anticoagulant; EAS, ectopic ACTH syndrome; HIT, heparin-induced thrombocytopenia; IPSS, inferior petrosal sinus sampling; LMWH, low-molecular-weight heparin; NOAC, non-vitamin K antagonist oral anticoagulant; UFC, urinary free cortisol; V/Q, ventilation/perfusion; VTE, venous thromboembolism.

“Not within the primary scope” indicates that the respective feature was outside the predefined focus of the publication and should not be interpreted as a limitation or omission of the proposed approach.

## 8 Practical conclusions and proposed thromboprophylaxis algorithm

### 8.1 Integrated clinical implications

CS is now firmly established as a high-risk prothrombotic condition, with VTE representing one of its most serious and potentially fatal complications. The available evidence consistently demonstrates that the highest thrombotic risk occurs in the peri-operative period, yet a substantial residual risk persists for weeks to months after biochemical remission (4, 8). Despite this, current management remains heterogeneous, and standardized, evidence-based protocols are lacking.

Three key principles emerge from the literature:

1. VTE risk in CS is both disease-driven and treatment-related.
2. Hypercoagulability persists beyond surgical cure.
3. Extended thromboprophylaxis is justified in most patients.

These observations support consideration of an approach that extends beyond standard short-term postoperative thromboprophylaxis.

### 8.2 Proposed clinical algorithm for VTE prophylaxis in CS

Based on registry data (ERCUSYN), observational studies, and the Delphi consensus 2025, the following stepwise approach is proposed (5, 9):

Step 1 – Risk assessment at diagnosis

Patients with confirmed endogenous CS may be considered at least at moderate risk of VTE, with additional clinical factors warranting classification as high risk. Additional high-risk features include:

- prior VTE,
- severe hypercortisolism (UFC >3–5× ULN),
- morbid obesity (BMI ≥35 kg/m²),
- immobility,
- malignancy or ectopic ACTH,
- estrogen therapy,
- markedly elevated FVIII (>150 IU/dL) or PAI-1.

If ≥1 high-risk factor is present → classify as high VTE risk.

Step 2 – Pre-operative phase

- Consider mechanical prophylaxis from the time of diagnosis.
- LMWH prophylaxis should be considered pre-operatively in moderate- to high-risk patients unless contraindicated.
- Consider screening duplex ultrasound of lower extremities in:
  ○ patients with previous VTE,
  ○ severe hypercortisolism,
  ○ prolonged immobility.
- Before initiating pharmacological thromboprophylaxis, postoperative bleeding risk should be reassessed, particularly after transsphenoidal surgery, and the timing of anticoagulation should be determined in collaboration with the treating neurosurgical team.

Step 3 – In-hospital peri-operative phase

- Consider combined mechanical and pharmacologic prophylaxis in most patients undergoing surgery.
- LMWH is generally preferred at standard prophylactic doses.
- Temporary interruption may be appropriate in patients with active bleeding or substantial surgical bleeding risk.

Step 4 – Post-discharge phase

- Consider extended thromboprophylaxis (4–12 weeks) in moderate- and high-risk patients, including many patients undergoing TSS or adrenalectomy, following individualized assessment of thrombotic and bleeding risk.
- Extend prophylaxis up to 3 months if:
  - biochemical remission is delayed,
  - FVIII, vWF or PAI-1 remain elevated,
  - mobility is reduced.
- Consider transition to DOAC after early postoperative phase in stable patients.
- Patients classified as high risk are more likely to benefit from early pharmacologic thromboprophylaxis and extended post-discharge prophylaxis (up to 12 weeks or longer), whereas lower-risk individuals may be treated according to standard postoperative protocols.

Step 5 – Reassessment after remission

- Reassess:
  - cortisol status,
  - FVIII, vWF, D-dimer,
  - clinical risk profile.
- Persistent laboratory evidence of hypercoagulability may support continuation of thromboprophylaxis or secondary prevention after individualized reassessment.
- If coagulation parameters normalize and no additional risk factors are present, discontinuation of anticoagulation after 3–6 months may be considered.

### 8.3 Safety considerations

- Bleeding risk must be assessed individually using clinical judgment rather than standard surgical scores alone.
- Particular caution is required in:
  - patients receiving high-dose glucocorticoid replacement,
  - those treated with CYP3A4-inducing steroids (e.g., dexamethasone) during DOAC therapy,
  - patients with postoperative hypopituitarism and fluctuating cortisol exposure.
- The decision to initiate pharmacological thromboprophylaxis should always balance thrombotic benefit against postoperative bleeding risk and ideally be made in collaboration with the treating neurosurgical team.

### 8.4 Final key messages

- CS should be regarded as a persistent prothrombotic state, not limited to the preoperative or hospitalized phase.
- Current evidence and expert consensus support consideration of in-hospital thromboprophylaxis and, in selected high-risk patients, extended post-discharge prophylaxis following individualized assessment of thrombotic and bleeding risk.
- The Delphi 2025 recommendations provide the best current guidance, but require prospective validation.
- Future management should evolve toward biomarker-driven, individualized prophylaxis strategies.
